# Positive Association of the Dietary n-6/n-3 PUFA Ratio with Fatty Liver in Mexican Adults

**DOI:** 10.3390/healthcare13212679

**Published:** 2025-10-23

**Authors:** Cristina Gutierrez-Osorio, Omar Ramos-Lopez

**Affiliations:** Faculty of Medicine and Psychology, Autonomous University of Baja California, Tijuana 22390, Baja California, Mexico; cgutierrez5@uabc.edu.mx

**Keywords:** n-6/n-3 ratio, fatty liver, obesity, Mexican adults

## Abstract

**Background:** The increase in obesity rates and related liver diseases has risen in recent years in Mexico. Dietary factors, such as the imbalance between n-6 and n-3 polyunsaturated fatty acids, have been associated with a higher risk of developing conditions such as fatty liver. The objective of this study was to analyse the influence of the dietary n-6/n-3 ratio on fatty liver in Mexican adults. **Methods**: This analytical cross-sectional study included 213 Mexican adults aged 18 to 65 years, of both genders. The dietary n-6/n-3 ratio was calculated using the Nutritionist Pro software. Participants were divided into two groups according to the median of their dietary n-6/n-3 intake ratio: “low” (<10.2:1) and “high” (≥10.2:1). Anthropometric and biochemical markers were evaluated using standardised methods. The hepatic steatosis index (HSI) was used as a surrogate marker of fatty liver. Multivariate logistic regression analyses were conducted to predict fatty liver based on HSI. **Results**: Overall, the mean dietary n-6/n-3 ratio was 12.75 in the general population. Higher HSI values were found in participants with a high n-6/n-3 ratio (*p* = 0.038). In the multivariate model, the n-6/n-3 ratio was positively associated with high HSI (OR = 1.48, 95% CI, 1.02, 1.99). **Conclusions**: This study concludes that a high n-6/n-3 ratio may contribute to the development of fatty liver in Mexican adults. These results highlight the importance of a balanced intake of fatty acids to prevent metabolic complications and improve public health.

## 1. Introduction

Non-alcoholic fatty liver disease (NAFLD) has become one of the leading causes of liver disease worldwide [1]. It is characterised by excessive accumulation of lipids, primarily triglycerides, in liver cells, and is unrelated to alcohol consumption [2]. Its development is closely associated with various metabolic factors such as obesity, insulin resistance, dyslipidaemia, and metabolic syndrome [3].

The global prevalence of NAFLD has been estimated at about 30%, with significant increases in recent years [4]. In Latin America, the average prevalence of NAFLD is around 24%, which may rise to 68% among high-risk populations with type 2 diabetes mellitus or obesity [5]. In Mexico, NAFLD represents a significant public health problem, with a predicted prevalence of up to 49.6% among adults based on national data [6].

Non-invasive diagnostic tools for detecting NAFLD include various clinical indices, such as the Hepatic Steatosis Index (HSI), which has been validated in several studies [7]. This index is calculated from routinely used clinical and biochemical parameters, allowing estimation of hepatic steatosis without the need for invasive procedures [8]. This tool constitutes a practical and low-cost alternative for population screening and follow-up in epidemiological studies or in clinical settings with limited access to advanced imaging methods [9].

Multiple dietary factors may contribute to the development of NAFLD, including a high n-6/n-3 polyunsaturated fatty acid (PUFA) ratio, which is a relevant nutritional indicator reflecting the balance between these two types of essential fats in the diet [10]. Overall, n-6/n-3 ratios ranging from 1:1 to 1:4 are considered healthy [11]. It has also been documented that the human body can maintain optimum health with an intake ratio of n-6/n-3 of 5:1 [12]. However, Westernised diets (including the current Mexican diet) are characterised by high consumption of n-6, which raises the n-6/n-3 ratio to a range of 10:1 to 20:1 [13]. Consequently, unbalanced n-6/n-3 ratios have been associated with an increased risk of metabolic, inflammatory, and liver diseases [14,15,16]. In this regard, it has been shown that individuals with NAFLD have a lower n-3 and higher n-6 PUFA dietary intake compared to healthy controls [17]. Similarly, a significant association between total PUFA intake and the occurrence of NAFLD has been documented in Chinese adults [18]. Consequently, a low n-6/n-3 dietary PUFA ratio has been evaluated as a treatment for fatty liver disease in obese patients, which ameliorated this metabolic phenotype, independent of weight loss [19].

NAFLD prevalence in Mexico is high and has been associated with unbalanced diets and low physical activity. Identifying specific modifiable factors, including diet, that contribute to this condition is important for prevention and for guiding personalised nutritional strategies for the precise management of NAFLD. The n-6/n-3 ratio has attracted interest due to its impact on metabolic health. However, there is still little evidence regarding its role in NAFLD status in Mexico. The aim of this study was to analyse the association of the dietary n-6/n-3 ratio with markers of fatty liver disease in Mexican adults.

## 2. Materials and Methods

### 2.1. Participants

This cross-sectional ancillary study was based on existing data from a previous study [20]. The population comprised 213 adults (18–65 years old), of both biological sexes, residing in Tijuana, Baja California, Mexico. Participants were randomly recruited at the Integral Healthcare Centre of the Autonomous University of Baja California (UABC) using convenience sampling. Individuals with established chronic diseases and/or undergoing pharmacological treatment (including diabetes, cardiovascular diseases, NAFLD, hormonal disorders, gastrointestinal problems, and cancer) were excluded, as were pregnant or lactating women, smokers, and alcohol drinkers (consuming more than 20 g/day and 40 g/day of ethanol for women and men, respectively).

The study protocol was approved by the Research Bioethics Committee of the Faculty of Medicine and Psychology of UABC (code: D235, approved on 22 October 2019). The research was conducted in accordance with the ethical principles for human research outlined in the Declaration of Helsinki. All participants voluntarily provided written informed consent.

### 2.2. Anthropometry

Height (cm) was measured using a stadiometer (Rochester Clinical Research, New York, NY, USA). Body weight (kg), percentage of body fat, and BMI (kg/m^2^) were assessed using a Tanita SC-331S body composition analyser (Tanita Corporation, Tokyo, Japan). Waist circumference (WC) was measured at the midpoint between the last rib and the top of the iliac crest; hip circumference (HC) was measured at the level of the greater trochanters using a non-stretchable tape with participants standing. The waist-to-hip ratio (WHR) was calculated by dividing WC by HC. Neck circumference (NC) was measured at the mid-neck, between the laryngeal prominence and the midcervical spine. All anthropometric measurements were performed by trained nutritionists.

Systolic and diastolic blood pressure were measured three times with an Omron HEM-7120 digital monitor (OMRON Corp., Kyoto, Japan), following international standards [21]. The body adiposity index (BAI) was estimated using the following formula: BAI = [HC (cm)/height (m)^1.5^] − 18 [22]. The visceral adiposity index (VAI) was estimated as VAI = (WC (cm)/(39.68 + (1.88 × BMI))) × (triglycerides/1.03) × (1.31/high-density lipoprotein cholesterol [HDL-c]) for males and VAI = (WC (cm)/(36.58 + (1.89 × BMI))) × (triglycerides/0.81) × (1.52/HDL-c) for females [23].

### 2.3. Dietary Evaluation

PUFA intake and macronutrient distributions were assessed using three 24 h recalls (two on weekdays and one on a weekend day) to obtain information on the type, quantity, and preparation of foods consumed. Trained personnel administered the recalls using food models and digital scales to improve the accuracy of portion estimation. This method has been previously validated in the Mexican population, supporting the reliability of the collected data [24]. Dietary records were analysed using Nutritionist Pro software (Axxya Systems, Stafford, TX, USA. Available on: https://nutritionistpro.com/, accessed on 31 August 2025), which allowed estimation of average PUFA intake and calculation of the n-6/n-3 dietary ratio as well as total energy and macronutrient distribution for each participant.

### 2.4. Biochemical Analysis

After a 12 h overnight fast, 10 mL of venous blood was collected by venepuncture and centrifuged for subsequent analysis. Fasting glucose, total cholesterol, triglycerides, high-density lipoprotein cholesterol (HDL-c), low-density lipoprotein cholesterol (LDL-c), alanine aminotransferase (ALT), aspartate aminotransferase (AST), and gamma-glutamyl transferase (GGT) concentrations were determined using commercial enzyme kits and a Mindray BS-200 automatic analyser (Mindray Medical International Ltd., Shenzhen, China). The triglyceride to HDL ratio was calculated by dividing triglyceride by HDL blood levels. The HSI [25] was calculated as a proxy for fatty liver according to the following formula: HSI = 8 × (ALT/AST) + BMI + 2 (if type 2 diabetes) + 2 (if female).

### 2.5. Statistical Analysis

The minimum sample size required for the low (*n* = 106) and high (*n* = 107) n-6/n-3 ratio groups was calculated to detect at least a 25% of difference in the average n-6/n-3 ratio between groups, achieving 80% statistical power at a 0.05 overall significance level. The normality of the main study variables (n-6/n-3 dietary ratio and fatty liver markers) was assessed using the Kolmogorov–Smirnov test (*p* > 0.05). Continuous variables were expressed as means ± standard deviations, while categorical variables were presented as number of cases and percentages. Participants were divided into two groups based on the median n-6/n-3 ratio in their diet and classified as “low” (<10.2:1) or “high” (≥10.2:1). Comparable n-6/n-3 ratio cut-offs (11.7:1) have been used in the Mexican population [26]. HSI ≥ 36 was considered a surrogate cut-off for NAFLD risk, as previously reported [27]. Thus, HSI was grouped as “low” (HSI < 36) or “high” (HSI ≥ 36). Differences in anthropometric and biochemical variables between study groups were analysed using Student’s *t*-test for independent samples. Differences in sex and frequencies of low and high groups were assessed using the chi-square test (χ^2^). Multivariate logistic regression analyses were conducted for predicting fatty liver based on HSI, and odds ratios (ORs) with 95% confidence intervals were reported. Bonferroni correction for multiple comparisons was applied. Statistical analyses were performed using IBM SPSS Statistics v20 (IBM Corp., Armonk, NY, USA), with a *p*-value < 0.05 considered statistically significant.

## 3. Results

Table 1 presents demographic, anthropometric, and nutritional characteristics by HSI group. Participants with high HSI were older and had greater body weight, BMI, body fat, WC, HC, WHR, NC, BAI, and VAI than those with low HSI (Table 1). Regarding the nutritional profile, participants with high HSI had higher caloric intake and a higher n-6/n-3 ratio than their counterparts.

Table 2 presents comparisons of biochemical variables by HSI group. Participants with high HSI had higher fasting glucose, total cholesterol, LDL-c, triglycerides, ALT, and GGT than those with low HSI. Conversely, individuals with high HSI had lower HDL-c values (Table 2).

Overall, the mean dietary n-6/n-3 ratio was 12.8:1. Table 3 presents demographic and anthropometric measurements by n-6/n-3 ratio group. No significant differences were observed between groups in BMI, body fat percentage, WC and HC, WHR, NC, SBP, DBP, or BAI (*p* > 0.05). However, VAI was significantly higher in the high n-6/n-3 ratio group (*p* = 0.044).

Table 4 presents comparisons of biochemical variables by n-6/n-3 ratio group. Participants with a high n-6/n-3 ratio had significantly lower HDL-c levels (*p* = 0.012) and a higher triglyceride/HDL ratio (*p* = 0.031). Similarly, HSI was significantly higher in the group with a high n-6/n-3 ratio (*p* = 0.038). No significant differences were observed between groups in glucose, total cholesterol, LDL-c, triglycerides, or the liver enzymes ALT, AST, and GGT.

**Figure 1:**
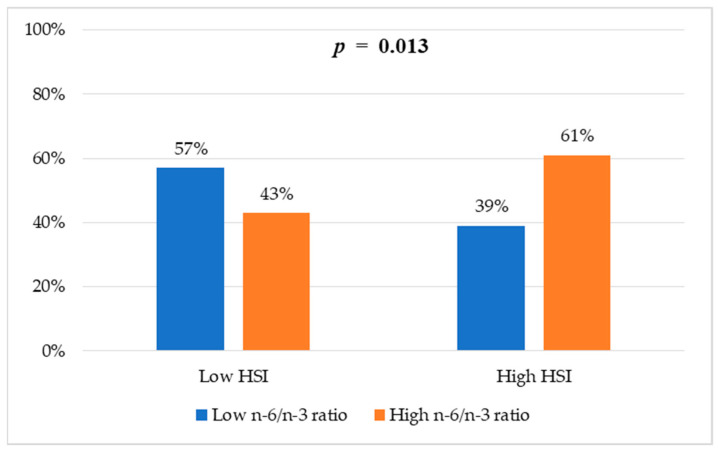
Frequencies of HSI categories according to n-6/n-3 ratio groups. Values are reported as percentages. Chi-square test was used to assess differences between groups.

Figure 1 shows the proportion of participants with and without risk of hepatic steatosis according to the HSI. Significant differences were observed between groups according to their n-6/n-3 fatty acid ratio (*p* = 0.013). In the group with a high n-6/n-3 ratio, 61% had a high HSI compared with 39% in the group with a low n-6/n-3 ratio.

Table 5 presents multivariate logistic regression analysis to predict fatty liver based on HSI. Age, sex, total energy intake, and n-6/n-3 ratio were significant risk factors for fatty liver. Overall, this model predicted fatty liver in 18% of cases (Table 5).

## 4. Discussion

NAFLD is currently one of the main causes of liver disease in Mexico and has been associated with metabolic and dietary disorders common in the Mexican population, including obesity, insulin resistance, dyslipidaemia, and type 2 diabetes [4,5]. In this study, participants with a high HSI (≥36) had greater adiposity (mainly abdominal obesity measurements) and more metabolic disorders (including glucose, lipid, and liver enzyme abnormalities) than those with a low HSI (<36). Our results are consistent with previous reports, in which participants with an HSI >36 had significantly higher values for obesity-related parameters, dyslipidaemia, insulin resistance, and inflammation in renal transplant recipients [28]. In fact, HSI ≥ 36 was significantly associated with features of metabolic syndrome and correlated with adiposity and inflammatory markers in patients with type 1 diabetes [29]. Moreover, higher levels of glucose, triglycerides, liver enzymes, BMI, and WC, as well as lower HDL levels, were observed in participants with metabolic syndrome and HSI ≥ 36 [30]. Overall, these results support the utility of HSI as a tool for diagnosing fatty liver [25,27] and characterising patients with excessive adiposity and metabolic disorders.

In this study, a high dietary n-6/n-3 ratio (≥10.2:1) was significantly associated with fatty liver compared to those with a low n-6/n-3 ratio (<10.2:1). The dietary n-6/n-3 ratio was also identified as a relevant predictor of fatty liver based on HSI in the multivariate logistic regression analyses after adjustment for age and sex. This association was supported by significant differences in adiposity markers (VAI) and lipid profiles, such as HDL-c and the triglyceride/HDL ratio, which were less favourable in the group with a high n-6/n-3 ratio. These findings are consistent with previous reports, where patients with NAFLD had a higher dietary n-6/n-3 ratio compared to controls [17]. Similarly, an increase in the dietary n-6/n-3 ratio resulted in a higher liver fibrosis score in a sample of Mexican-origin Hispanic adults with overweight or obesity [31]. A high dietary n-6/n-3 ratio was also positively associated with excessive adiposity, WC, and insulin resistance in Mexican adults [26]. Furthermore, a high n-6/n-3 ratio was associated with low HDL levels, while reducing this ratio improved the lipid profile in adults with dyslipidaemia [32].

The underlying mechanisms linking an unbalanced dietary n-6/n-3 PUFA ratio with NAFLD and metabolic syndrome features involve increased production of pro-inflammatory molecules and impaired regulation of hepatic and adipose function [33]. Excessive hepatic uptake of n-6 PUFA promotes the formation and accumulation of oxidised linoleic acid metabolites, which are known risk factors for the development of fatty liver and progression to hepatic fibrosis [34]. Notably, a high n-6/n-3 PUFA ratio may contribute to fatty liver development due to impaired regulation of liver lipid metabolism and related oxidative processes [35]. Additionally, the n-6/n-3 ratio plays an important role in promoting excessive adiposity through eicosanoid metabolite production, hyperactivity of the cannabinoid system, adipogenesis, browning of adipose tissue, lipid metabolism, the brain–gut-adipose tissue axis, and systemic inflammation, which can be reversed with increased intake of n-3 fatty acids [36].

In this context, several experimental studies have evaluated the effects of n-6/n-3 fatty acids on liver health. For example, C57BL/6J mice fed a high-fat diet (HFD) plus n-6 PUFAs for 10 weeks developed insulin resistance and fatty liver through upregulation of the expression of lipogenesis-related genes in the liver, whereas the opposite effects were observed in HFD mice supplemented with n-3 PUFAs [37]. Similarly, C57BL/6J mice fed an HFD enriched with n-3 fatty acids, with a 5:1 n-6/n-3 ratio over 17 weeks, showed improved lipid and liver markers and inflammation-related gene expression compared to those with a 30:1 n-6/n-3 ratio [38]. Moreover, histopathological analyses revealed that mice fed a diet enriched with n-6 PUFA over 20 weeks had a significant increase in macrovesicular steatosis, apoptotic hepatocytes, and decreased glycogen storage compared to mice fed a diet enriched with n-3 PUFA [39]. Interestingly, a decrease in the tissue n-3/n-6 PUFA ratio correlated with steatosis and hypercholesterolaemia as well as an increase in hepatic cholesteryl ester and triglyceride content in murine livers [40]. In fact, n-3 PUFA depletion in liver phospholipids led to activation of sterol regulatory element binding protein-1c (SREBP-1c) and lipogenesis, which contributed to hepatic steatosis development in C57BL/6J mice [41]. Accordingly, an n-3-enriched diet with an n-6/n-3 ratio of 4:1 reversed HFD-induced NAFLD by reducing the hepatic impairment of lipid homeostasis, oxidative stress, and the inflammatory responses in ApoE-/- mice [42]. In addition, an n-3-enriched diet counteracted the development of HFD-induced fatty liver in male C57BL/6J mice, which was related to modulation of lipogenesis-related gene expression in the liver [43]. The beneficial effects of n-3 PUFA on reducing the severity of the lipid metabolism disorder and liver damage in C57BL/6 J mice may also be linked to upregulation of the *Fra1* gene and attenuated activity of c-Jun and c-Fos [44].

The dietary n-6/n-3 ratio has been used as a valuable nutritional tool to evaluate the health status as well as to estimate the risk of developing chronic diseases such as cardiovascular disease, cancer, and inflammatory and autoimmune diseases [45]. The mean n-6/n-3 ratio observed in this sample (12.8:1) shows an unbalanced dietary pattern, which is similar to previous reports (14:1) in the Mexican population [26] and exceeds the proportions considered as healthy (1:1 to 4:1). Also, the n-6/n-3 ratio cutoffs (10.2:1) used in this study are comparable to those used previously in Mexicans (11.7:1), which were associated with obesity and a worse metabolic profile [26]. These dietary trends are probably related to the constant use of vegetable oils rich in linoleic acid, such as corn, sunflower, and soybean, which are characteristic of the Mexican diet, highlighting the need to promote greater consumption of dietary sources of omega-3, such as oily fish, salmon, sardines, and oilseeds [46]. From an evolutionary point of view, our findings are also consistent with the current nutritional pattern of Western diets, which are deficient in n-3 fatty acids and have excessive amounts of n-6 fatty acids compared with the diet on which human beings evolved, resulting in large increases in the n-6/n-3 ratio from 1:1 during evolution to 20:1 today or even higher [45].

The results of this study support the hypothesis that an imbalance in PUFA intake, particularly a high n-6/n-3 ratio, could represent a dietary risk factor for developing NAFLD and cardiometabolic disorders, being used in routine nutritional assessment [16]. However, some limitations should be considered. The cross-sectional design of this study precludes establishing definitive causal relationships between the n-6/n-3 ratio and fatty liver, but our results provide important findings for the design of further research in independent cohorts to assess the impact of the dietary n-6/n-3 ratio on liver disease prevention and management. Indeed, validation in controlled experimental settings (animal or in vitro studies) would strengthen the findings and provide more mechanistic support for the observed associations. Also, fatty acid intake was estimated using 24 h recalls, which may introduce memory bias and lead to reporting errors, as well as the fact that this tool may not accurately represent long-term dietary habits [47]. Nevertheless, they are relatively low-burden for respondents and can be conducted quickly and cost-effectively, and comparable studies have been performed using this nutritional instrument to estimate the intakes of macro- and micronutrients and their relationships with metabolic phenotypes in the Mexican population [24]. Another limitation of this study is that fatty liver diagnosis was made based on markers rather than clinical diagnosis, although HSI has proven to be a useful and reliable surrogate tool for fatty screening [25,27]. In addition, this fatty liver marker has been used in similar studies analysing associations between diet and liver status [20,48]. Furthermore, this research was conducted on individuals who were apparently healthy and were assumed to have no chronic illness and not be taking medication regularly; therefore, further case–control studies are needed to validate our results. Nonetheless, our results may allow early identification of individuals at high risk of developing fatty liver based on dietary n-6/n-3 ratio.

## 5. Conclusions

In conclusion, this study suggests that a high n-6/n-3 ratio may contribute to the development of fatty liver in Mexican adults. These results highlight the importance of a balanced intake of fatty acids to prevent metabolic complications and improve public health. Considering the n-6/n-3 ratio as a dietary marker in assessing metabolic risk, it could represent a useful tool for the management of NAFLD and its complications in the Mexican population.

## Figures and Tables

**Table 1:** Demographic, anthropometric, and nutritional characteristics according to HSI groups.

Variable	Low HSI (*n* = 132)	High HSI (*n* = 81)	*p* Value
*Demographics*			
Age (years)	35.2 ± 12.1	41.5 ± 11.3	**<0.001**
Sex (F/M)	98/32	34/49	**<0.001**
*Anthropometrics*			
Weight (kg)	66.3 ± 10.6	91.9 ± 16.0	**<0.001**
BMI (kg/m^2^)	24.9 ± 3.8	33.0 ± 4.7	**<0.001**
Body fat (%)	32.6 ± 8.6	39.6 ± 8.1	**<0.001**
WC (cm)	82.1 ± 9.7	102.9 ± 11.4	**<0.001**
HC (cm)	100.2 ± 7.1	113.8 ± 9.7	**<0.001**
WHR	0.8 ± 0.1	0.9 ± 0.1	**<0.001**
NC (cm)	34.6 ± 6.2	39.6 ± 4.2	**<0.001**
SBP (mmHg)	120.9 ± 16.4	119.7 ± 20.3	0.647
DBP (mmHg)	78.4 ± 10.2	79.0 ± 9.7	0.722
BAI	22.9 ± 4.5	27.6 ± 4.7	**<0.001**
VAI	3.6 ± 2.8	5.3 ± 3.3	**<0.001**
*Nutritional profile*			
Total calories (kcal)	1896 ± 678	2339 ± 1047	**<0.001**
Carbohydrates (%)	42.7 ± 9.9	41.7 ± 8.7	0.458
Proteins (%)	19.1 ± 5.7	19.2 ± 5.6	0.975
Fat (%)	37.4 ± 8.0	37.8 ± 8.5	0.729
Ratio n-6/n-3	10.4 ± 4.2	12.1 ± 4.9	**0.012**

Values are presented as means ± standard deviations, which were adjusted by age, and sex. BMI: body mass index; WC: waist circumference; HC: hip circumference; WHR = waist-hip ratio; NC: neck circumference; SBP: systolic blood pressure; DBP: diastolic blood pressure; BAI: body adiposity index; VAI: visceral adiposity index. Student’s *t*-tests and chi-square tests were used to assess differences between groups for continuous and categorical variables, respectively. *p* values in bold indicate statistically significant differences (*p* < 0.05).

**Table 2:** Comparison of lipid and liver biomarkers according to HSI groups.

Variable	Low HSI (*n* = 132)	High HSI (*n* = 83)	*p* Value
Fasting glucose (mg·100 mL^−1^)	92.6 ± 10.8	98.6 ± 14.3	**0.001**
Total cholesterol (mg·100 mL^−1^)	186.9 ± 38.8	200.1 ± 37.0	**0.015**
LDL-c (mg·100 mL^−1^)	118.8 ± 34.9	135.1 ± 31.5	**0.001**
HDL-c (mg·100 mL^−1^)	48.7 ± 13.5	40.6 ± 11.2	**<0.001**
Triglycerides (mg·100 mL^−1^)	92.9 ± 56.6	131.2 ± 62.5	**<0.001**
Triglyceride/HDL ratio	2.1 ± 1.5	3.9 ± 2.9	**<0.001**
ALT (IU·1000 mL^−1^)	20.1 ± 10.7	40.1 ± 21.8	**<0.001**
AST (IU·1000 mL^−1^)	36.6 ± 24.2	40.3 ± 10.7	0.201
GGT (IU·1000 mL^−1^)	15.1 ± 9.8	24.6 ± 17.6	**<0.001**

Values are presented as means ± standard deviations, which were adjusted by age, and sex. ALT: alanine aminotransferase; AST: aspartate aminotransferase; GGT: gamma glutamyl transferase; HSI: hepatic steatosis index. Student’s *t*-tests were used to assess differences between groups. *p* values in bold indicate statistically significant differences (*p* < 0.05).

**Table 3:** Comparison of demographic, anthropometric, and nutritional measurements according to n-6/n-3 ratio groups.

Variable	Low n-6/n-3 Ratio (*n* = 106)	High n-6/n-3 Ratio (*n* = 107)	*p* Value
*Demographics*			
Age (years)	37.4 ± 12.8	37.7 ± 12.1	0.877
Sex (F/M)	62/44	70/37	0.301
*Anthropometrics*			
Weight (kg)	74.1 ± 16.6	78.5 ± 19.1	0.080
BMI (kg/m^2^)	27.6 ± 5.0	28.6 ± 6.4	0.185
Body fat (%)	35.6 ± 8.8	34.1 ± 9.5	0.431
WC (cm)	88.7 ± 13.4	91.4 ± 15.5	0.186
HC (cm)	104.8 ± 10.0	106.1 ± 11.0	0.344
WHR	0.8 ± 0.1	0.9 ± 0.1	0.275
NC (cm)	36.2 ± 7.0	36.8 ± 4.9	0.477
SBP (mmHg)	120.0 ± 18.8	121.0 ± 17.4	0.713
DBP (mmHg)	77.3 ± 9.1	78.8 ± 13.2	0.347
BAI	24.4 ± 5.7	25.2 ± 4.6	0.251
VAI	3.8 ± 2.9	4.7 ± 3.3	**0.044**
*Nutritional profile*			
Total calories (kcal)	1985 ± 941	2132 ± 780	0.164
Carbohydrates (%)	43.3 ± 9.3	41.3 ± 9.6	0.134
Proteins (%)	19.3 ± 4.7	19.1 ± 6.3	0.818
Fats (%)	36.2 ± 8.3	38.8 ± 7.7	**0.024**

Values are presented as means ± standard deviations, which were adjusted by age, and sex. BMI: body mass index; WC: waist circumference; HC: hip circumference; WHR: waist-hip ratio; NC: neck circumference; SBP: systolic blood pressure; DBP: diastolic blood pressure; BAI: body adiposity index; VAI: visceral adiposity index. Student’s *t*-tests and chi-square tests were used to assess differences between groups for continuous and categorical variables, respectively. Values in bold indicate statistically significant differences (*p* < 0.05).

**Table 4:** Comparison of lipid and liver biomarkers according to n-6/n-3 ratio groups.

Variable	Low n-6/n-3 Ratio (*n* = 106)	High n-6/n-3 Ratio (*n* = 107)	*p* Value
Fasting glucose (mg·100 mL^−1^)	96.5 ± 13.8	93.2 ± 11.1	0.061
Total cholesterol (mg·100 mL^−1^)	194.5 ± 37.8	190.2 ± 39.1	0.413
LDL-c (mg·100 mL^−1^)	127.3 ± 33.8	123.4 ± 35.1	0.416
HDL-c (mg·100 mL^−1^)	48.0 ± 13.9	43.5 ± 12.4	**0.012**
Triglycerides (mg·100 mL^−1^)	102.1 ± 56.4	113.9 ± 66.0	0.163
Triglyceride/HDL ratio	2.37 ± 1.6	3.2 ± 3.6	**0.031**
ALT (IU·1000 mL^−1^)	26.2 ± 16.2	33.4 ± 39.5	0.086
AST (IU·1000 mL^−1^)	36.1 ± 8.7	40.3 ± 26.9	0.119
GGT (IU·1000 mL^−1^)	17.7 ± 11.2	20.3 ± 16.5	0.176
HSI	33.8 ± 6.7	35.9 ± 7.5	**0.038**

Values are presented as means ± standard deviations, which were adjusted by age, and sex. ALT: alanine aminotransferase; AST: aspartate aminotransferase; GGT: gamma glutamyl transferase; HSI: hepatic steatosis index. Student’s *t*-tests were used to assess differences between groups. *p* values in bold indicate statistically significant differences (*p* < 0.05).

**Table 5:** Multivariate logistic regression analyses using demographic, biochemical, and nutritional features to predict fatty liver based on HSI.

Variable	OR (95% CI)	*p* Value
Age (years)	1.04 (1.01, 1.07)	**0.003**
Sex (M)	2.37 (1.15, 4.85)	**0.018**
Fasting glucose (mg·100 mL^−1^)	1.02 (0.98, 1.05)	0.121
Total cholesterol (mg·100 mL^−1^)	1.01 (0.98, 1.02)	0.083
Total energy (Kcal/d)	1.01 (1.0, 1.01)	**0.007**
n-6/n-3 ratio	1.48 (1.02, 1.99)	**0.007**
Adjusted R^2^	0.18	**0.012**

Values are reported as Odds Ratios (OR), with 95% confidence intervals (CI). Bold numbers indicate *p* < 0.05. *p* values are corrected by Bonferroni test for multiple comparisons.

## Data Availability

The original contributions presented in this study are included in the article. Further inquiries can be directed to the corresponding author.
